# Serum CHI3L1 Levels Predict Overall Survival of Hepatocellular Carcinoma Patients after Hepatectomy

**DOI:** 10.7150/jca.100791

**Published:** 2024-10-14

**Authors:** Yanji Jiang, Wenfeng Gong, Yingchun Liu, Zihan Zhou, Xiumei Liang, Qiuling Lin, Moqin Qiu, Biaoyang Lin, Xiaoqiang Qiu, Hongping Yu

**Affiliations:** 1 Department of Epidemiology and Health Statistics, School of Public Health, Guangxi Medical University, Nanning, Guangxi, 530021, China.; 2 Department of Scientific Research, Guangxi Medical University Cancer Hospital, Nanning, Guangxi, 530021, China.; 3 Hepatobiliary Surgery Department, Guangxi Medical University Cancer Hospital, Nanning, Guangxi, 530021, China.; 4 Department of Experimental Research, Guangxi Medical University Cancer Hospital, Nanning, Guangxi, 530021, China.; 5 Department of Cancer Prevention and Control, Guangxi Medical University Cancer Hospital, Nanning, Guangxi, 530021, China.; 6 Department of Disease Process Management, Guangxi Medical University Cancer Hospital, Nanning, Guangxi, 530021, China.; 7 Department of Clinical Research, Guangxi Medical University Cancer Hospital, Nanning, Guangxi, 530021, China.; 8 Department of Respiratory Oncology, Guangxi Medical University Cancer Hospital, Nanning, Guangxi, 530021, China; 9 Zhejiang University, Zhejiang-California International Nanosystems Institute (ZCNI) Proprium Research Center, Hangzhou, Zhejiang, 310058, China; 10 University of Washington School of Medicine, Seattle, WA, 98195, USA.; 11 Key Laboratory of Early Prevention and Treatment for Regional High-Frequency Tumor (Guangxi Medical University), Ministry of Education, Nanning, Guangxi, 530021, China.; 12 Key Cultivated Laboratory of Cancer Molecular Medicine, Health Commission of Guangxi Zhuang Autonomous Region, Nanning, Guangxi, 530021, China.

**Keywords:** Hepatocellular carcinoma, CHI3L1, Nomogram, Overall survival

## Abstract

**Objective:** The Chitinase 3-like protein 1 (CHI3L1) is currently used as a biomarker for the diagnosis of liver fibrosis. However, its prognostic value for hepatocellular carcinoma (HCC) patients remains controversial. In this study, we aimed to investigate the prognostic value of the CHI3L1 in HCC patients after hepatectomy.

**Methods:** In total, 753 HCC patients who underwent curative hepatectomy between January 2017 to August 2021 were retrospectively recruited. The probability of overall survival (OS) was evaluated by the Kaplan-Meier method and compared between groups using the log-rank test. Cox proportional hazard regression analysis was used to determine the independent prognostic factors. A prognostic nomogram was constructed for further examine the clinical utility of CHI3L1 in HCC.

**Results:** Kaplan-Meier analysis revealed that elevated serum CHI3L1 levels were associated with worse overall survival of HCC patients. Multivariate Cox regression analysis showed that the high-CHI3L1 group (≥198.94 ng/ml) was associated with a shorter survival time compared with that in the low-CHI3L1 group (< 198.94 ng/ml) after adjustment for potential confounding factors (HR =1.43, 95% CI = 1.05-1.94, *P* = 0.024). Additionally, the nomogram had sufficient calibration and discriminatory power in the training cohort, with C-indexes of 0.723 (95% CI: 0.673-0.772). The validation cohort showed similar results. Finally, we demonstrated that the AUC of the nomogram was 0.752 (95% CI: 0.683-0.821), which had better predictive ability than AFP (AUC: 0.644, 95% CI: 0.577-0.711).

**Conclusion:** Our results confirmed that the CHI3L1 could serve as an independent predictor for OS in HCC patients after hepatectomy. The nomogram showed a good performance in prognosis prediction of HCC.

## Introduction

Primary liver cancer (PLC) is a common malignancy that ranks sixth among all types of cancer. PLC is the third leading cause of cancer death worldwide 1. By 2025, liver cancer is estimated to affect more than 1 million people worldwide each year 2. The most common histologic subtype of liver cancer, hepatocellular carcinoma (HCC), accounts for 75%∼85% of the cases 1. Hepatectomy is an established curative treatment for patients with HCC, particularly for small HCC. However, the 5-year overall survival (OS) rate of patients with HCC is very unsatisfactory, remaining less than 50% after radical resection 3. Thus, it is imperative that novel biomarkers are identified to accurately predict the survival and to improve the management of HCC patients.

CHI3L1 (Chitinase 3-like protein 1, or YKL-40) is a 40-kDa secreted glycoprotein with a highly conserved heparin and chitin-binding lectin, belonging to the mammalian chitinase family 4. It is now well-established that CHI3L1 is endogenously expressed in a wide variety of cells, including neutrophils, macrophages, differentiated smooth muscle cell, endothelial cells, and tumor cells 5. Up to now, CHI3L1 is known to be stimulated by mediators such as interleukin (IL) -13, IL-6, IL-1β, and IFN-γ 6. It is also a fibroblast growth factor that is highly enriched in liver and significantly correlated with the degree of liver fibrosis 7. Intriguingly, previous studies have revealed that CHI3L1 could regulate various biological and cellular processes, including angiogenesis, inflammation, tissue remodeling, and is involved in the development of cancers 8-11. In fact, CHI3L1 is elevated at both the mRNA and the protein levels in a variety of cancers and in many animal tumor models, and its levels were correlated with the stages and the outcomes of multiple types of primary and secondary carcinomas 12-16. Consequently, CHI3L1 has been established as a promising biomarker for diagnosing different diseases and for prognosis. Studies have found that the HCC patients with higher CHI3L1 had significantly lower rates of OS and disease-free survival (DFS) than those with lower levels of CHI3L1, suggesting that CHI3L1 is an independent predictor for survival in HCC patients 17,18. However, these associations remain controversial since some other studies have found no relationship between CHI3L1 overexpression and survival in patients with liver cancer 19. In addition, it is worthwhile to study whether the combined use of CHI3L1 and other clinical indicators can improve the accuracy of prognosis prediction of HCC patients.

Here, we conducted this retrospective study to evaluate the prognostic value of the CHI3L1, alone or with other clinical indicators, in predicting OS of HCC patients undergoing hepatectomy. In the end, we developed and validated a prognostic nomogram for predicting OS of HCC patients by integrating serum CHI3L1 level and other independent prognostic factors.

## Materials and Methods

### Patient selection

Between January 2017 to August 2021, 753 patients diagnosed with HCC were recruited from the Guangxi Medical University Cancer Hospital. The selection criteria were as follows: (1) pathological diagnosis of HCC; (2) age from 18 to 85 years; (3) underwent complete surgical resection. Patients meeting the following criteria were excluded: (1) HCC with distant metastasis; (2) preoperative treatment performed with chemotherapy, radiotherapy, or transcatheter arterial chemoembolization; (3) no follow-up or incomplete clinical data. This study was approved by the Institutional Review Board of the Guangxi Medical University Cancer Hospital (reference no. LW2024028). All patients provided written informed consent.

### Sample collection and assay of serum CHI3L1 level

Peripheral blood samples were collected before surgery without any treatment and centrifuged at 3000r/min for 10 min. The serum was separated from plasma and then stored at -80°C. Serum CHI3L1 levels in HCC patients were measured by enzyme linked immunosorbent assay (ELISA). The test kits were provided by Hangzhou Proprium Biotech Company Limited (Hangzhou, China). The analyses were carried out following the manufacturer's instructions.

### Data collection and follow-up

Based on hospital electronic medical records, we collected the information of HCC patients underwent hepatectomy retrospectively. The characteristics of the patients included their age, sex, height, weight, body mass index (BMI), smoking status, alcohol consumption, history of hypertension and diabetes mellitus (DM), and the family history of liver cancer. The clinicopathological parameters included tumor number, tumor size, Child-Pugh grade, cirrhosis, and Barcelona Clinic Liver Cancer (BCLC) stage. According to the Chinese Guideline for surgical treatment for HCC patients 20, some patients with BCLC stage B or C stage were ultimately included in the current study. The laboratory examinations included hepatitis B surface antigen (HBsAg), hepatitis B e antigen (HBeAg), alpha-foetoprotein (AFP), des-gamma-carboxy prothrombin (DCP), neutrophil-to-lymphocyte ratio (NLR), and international normalized ratio (INR).

The patients were followed up every three months following surgery for the first two years, and then every 6 months thereafter via phone calls until the death of the patient or till August 2023. Patients' overall survival (OS) was the primary endpoint of the study. Follow-up time was defined as the time between the date of hepatectomy and the date of death or the last effective follow-up.

### Statistical analysis

Categorical variables were expressed as N (%), and chi-square test was used to test differences in the distribution of covariates between groups. Time-dependent receive operating characteristic (ROC) curve analysis was used to determine the optimal cutoff value of continuous variable CHI3L1, NLR, and INR. The survival curves of CHI3L1 were calculated using the Kaplan-Meier method and assessed using the log-rank test. We used univariate Cox regression analysis to identify potential risk factors associated with OS. Variables with *P* < 0.05 in univariable analysis were entered into multivariable Cox regression model, and then backward stepwise selection was performed to select independent risk factors. Results were reported using the *P* values, and the hazard ratio (HR) with their respective 95% CI.

To study whether CHI3L1 in combination with other clinical parameters performs better than CHI3L1 alone in predicting OS in HCC patients undergoing hepatectomy, we constructed a nomogram. We randomly divided all HCC patients into the training (n = 527) and the validation (n = 226) cohorts with a ratio of 7 to 3. A nomogram for predicting 1-, 3-, and 5-year survival rates in HCC patients was constructed combining CHI3L1 with other independent risk factors. The performance of the nomogram in predicting OS was evaluated by Harrell's concordance index (C-index) and the area under the curve (AUC) of the ROC curves. The value of the C-index and AUC range from 0.5 (no discrimination at all) to 1.0 (perfect discrimination). Additionally, we used calibration curves to compare the nomogram-predicted survival rates with the actual survival rates in the 1-, 3-, and 5-year time periods. Finally, according to the median of risk score, all patients were divided into the high-risk and low-risk groups, and the prognostic differences between the high-risk and low-risk groups were analyzed by log-rank test. SPSS 25.0 (IBM, Chicago, IL, USA) and R software (version 4.3.2) were used for statistical analysis of the data. A two tailed *P* value < 0.05 was considered statistically significant.

## Results

### CHI3L1 was associated significantly with age, sex, tumor size, the HCC biomarker DCP, and NLR

**Figure 1:**
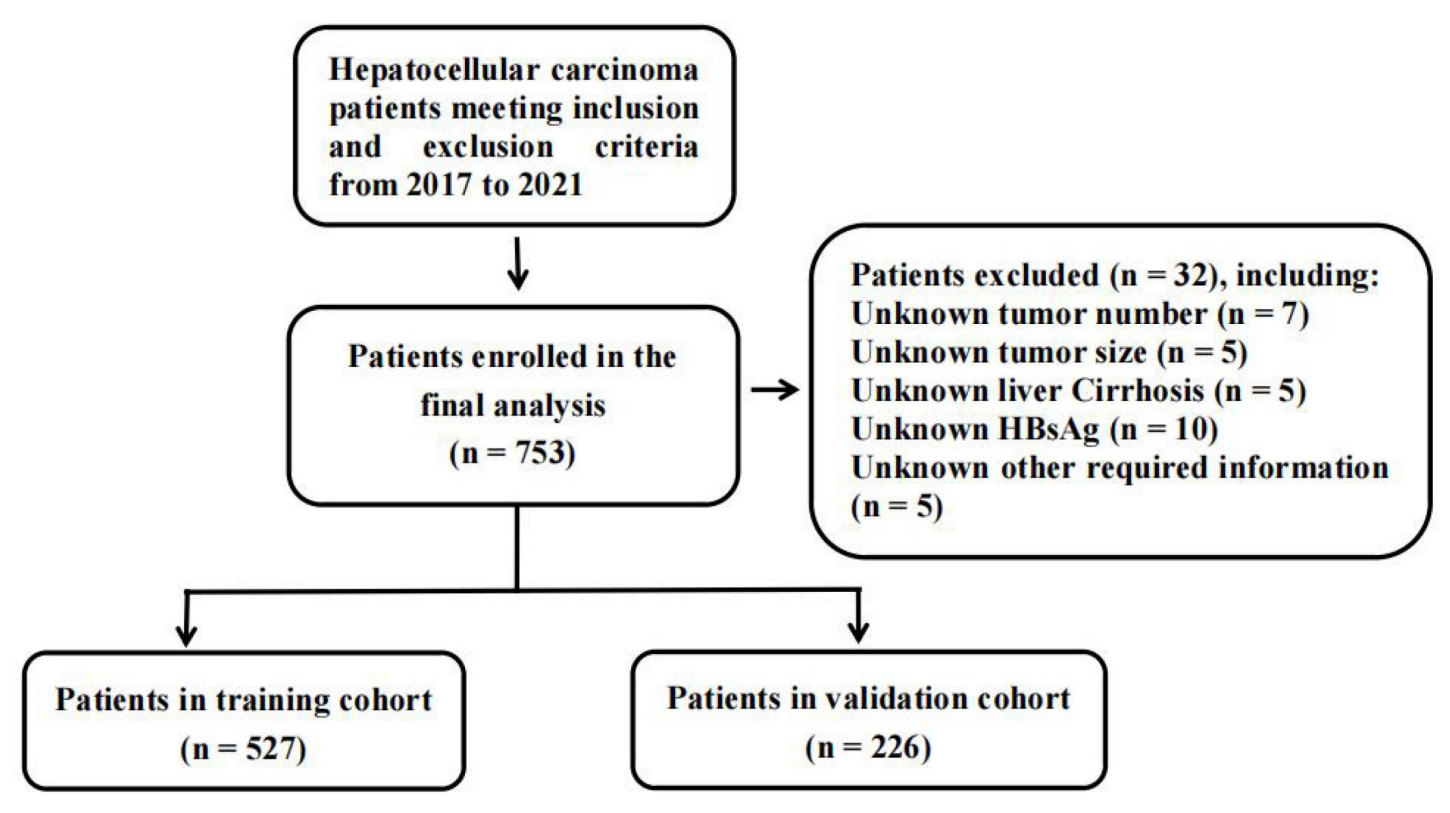
A flowchart for inclusion and exclusion of HCC patients in the study.

A total of 753 HCC patients were included for this study and a flowchart showing the inclusion and exclusion criteria was shown in **Figure 1**. The clinical and pathologic characteristics grouped by CHI3L1 were summarized in **Table 1**. The majority of patients (87.6%) were males, while the minority (12.4%) were females. The median age of the patients was 52 years (range, 24 - 83 years). We found that CHI3L1 was significantly associated with age (*P* < 0.001), sex (*P* = 0.002), tumor size (*P* < 0.001), the HCC biomarker DCP (*P* < 0.001), and NLR (*P* = 0.003) (**Table 1**). Patients were divided into the high- and the low-CHI3L1 expression groups by the optimal cutoff value of 198.94 for CHI3L1 based on the time-dependent ROC curve analysis. 468 patients (62.2%) and 285 patients (37.8%) fell into the low-CHI3L1 group (<198.94 ng/ml) and high-CHI3L1 group (≥198.94 ng/ml), respectively.

### High-CHI3L1 level predicted shorter OS of HCC patients

**Figure 2:**
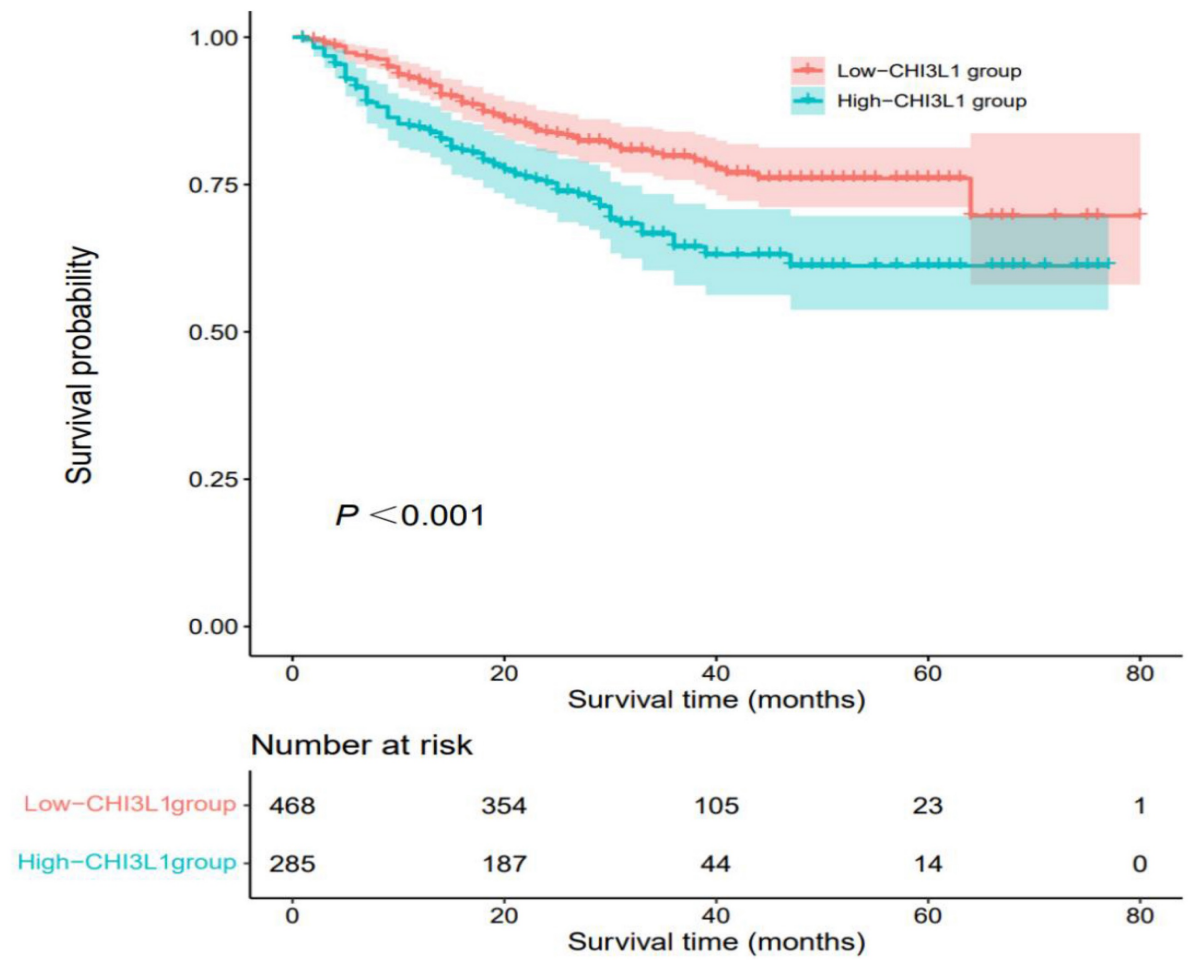
Kaplan-Meier analyses for OS of HCC patients based on preoperative CHI3L1.

The median OS was 27 months (range, 1 month to 80 months) and death occurred in 170 (22.6%) patients. According to Kaplan-Meier analysis, we found that the OS of HCC patients in the high-CHI3L1 group was shorter than that in the low-CHI3L1 group (*P* < 0.001, **Figure 2**). Other variables associated with OS in study cohorts analyzed by univariate and multivariate Cox regression analyses were summarized in **Table 2**. The high-CHI3L1 group (≥198.94 ng/ml) had a shorter survival time compared with the low-CHI3L1 group (< 198.94 ng/ml) after adjustment for potential confounding factors (HR =1.43, 95% CI = 1.05-1.94, *P* = 0.024). We also found that the tumor size (HR =1.61, 95% CI = 1.10-2.35, *P* = 0.013), the biomarker AFP (HR =1.70, 95% CI = 1.25-2.31, *P* < 0.001) and DCP (HR =1.67, 95% CI = 1.06-2.62, *P* = 0.026), and NLR (HR =2.07, 95% CI = 1.47-2.91, *P* < 0.001), INR (HR =1.58, 95% CI = 1.16-2.14, *P* = 0.003) were all independent prognostic factors for HCC survival.

### Construction of a nomogram for improved performance in OS prediction for HCC patients and its validation

We divided 753 HCC patients randomly divided into the training (n = 527) and the validation (n = 226) cohorts. The clinical and pathologic characteristics of the HCC patients in the study cohort are described in **Table S1**. Clearly, there were no differences in the baseline characteristics between the training cohort and the validation cohort, except for the tumor number (*P =* 0.017). Univariate and multivariate Cox regression analyses were conducted in the training cohort and the result of OS-related variables were summarized in **Table 3**. All significant prognostic factors for OS (*P* < 0.05) in the univariable analysis were enrolled in the multivariable analysis. In addition, considering that the tumor number (*P* = 0.050) is closely related to the survival of HCC, it is also included in the multivariate model. Finally, multivariate regression analysis revealed that the tumor number (HR =1.66, 95% CI = 1.08-2.57, *P* = 0.022), the tumor size (HR =1.61, 95% CI = 1.08-2.57, *P* = 0.013), the traditional HCC biomarker AFP (HR =1.97, 95% CI = 1.35-2.87, *P* < 0.001), NLR (HR =2.34, 95% CI = 1.55-3.52, *P* < 0.001), INR (HR =1.45, 95% CI = 1.00-2.49, *P* = 0.041) and CHI3L1 (HR =1.46, 95% CI = 1.00-2.13, *P* = 0.049) were all independent prognostic factors.

**Figure 3:**
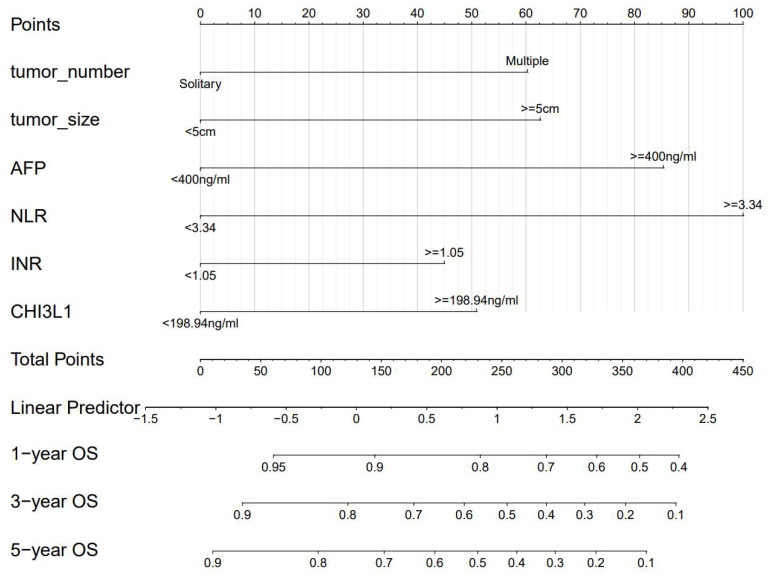
Nomogram for OS in HCC patients after hepatectomy. OS, overall survival; AFP, alpha-fetoprotein; DCP, des-gamma-carboxy prothrombin; NLR, neutrophil-to-lymphocyte ratio; INR, international normalized ratio.

**Figure 4:**
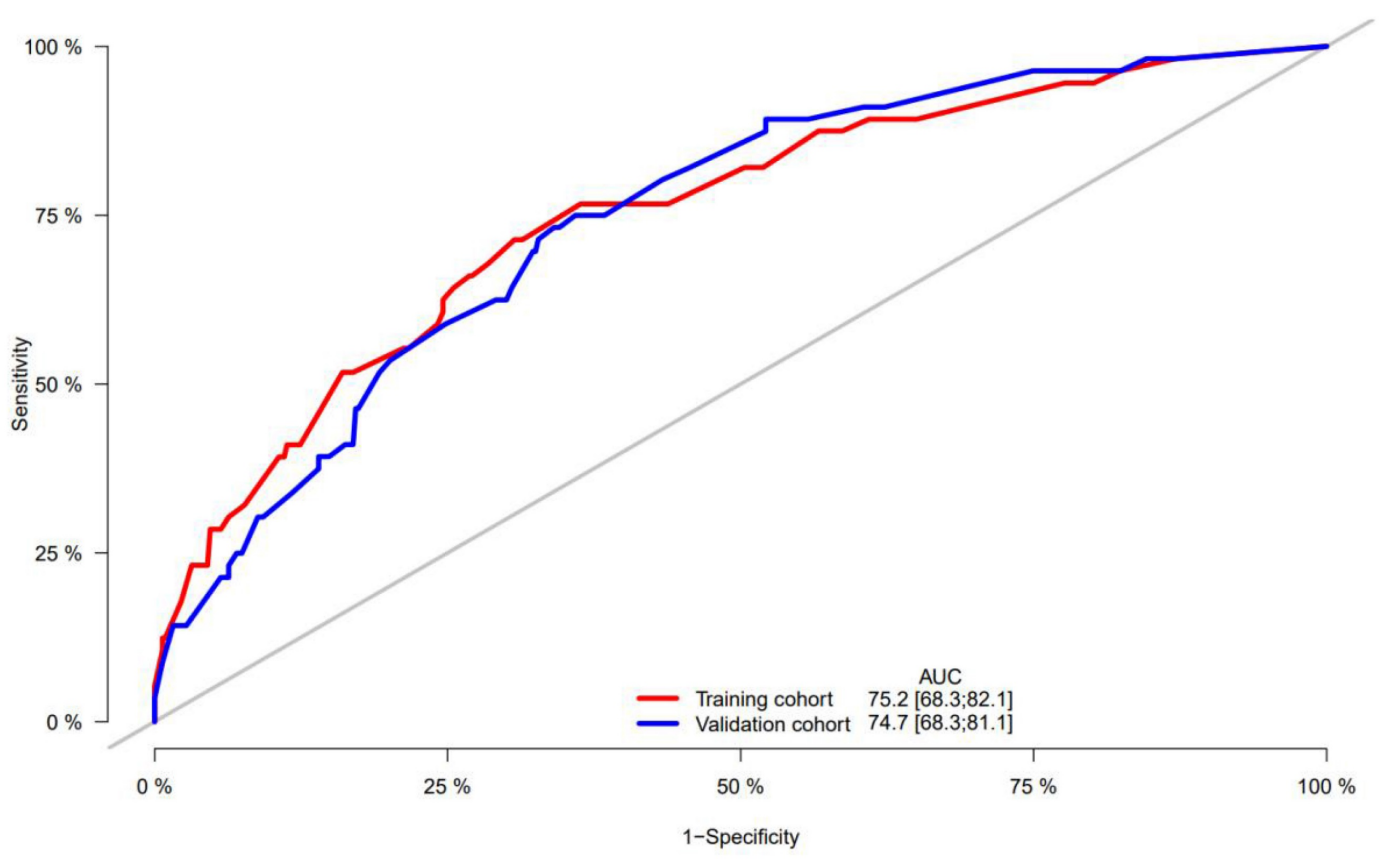
ROC curve of the nomogram in the training and validation cohorts.

**Figure 5:**
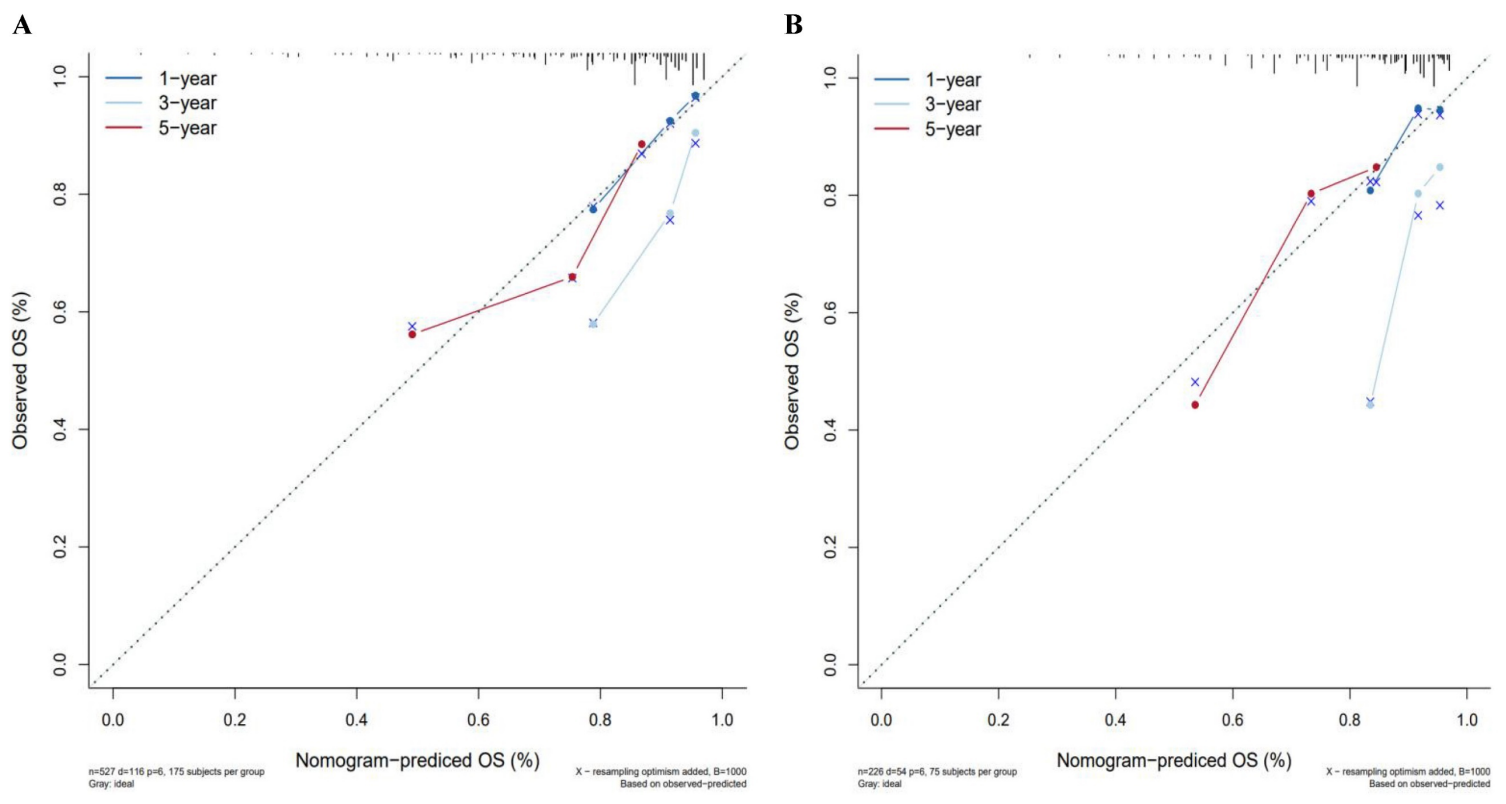
Calibration curves for predicting HCC patient survival in the training and validation cohorts. (A) 1-, 3-, and 5-year survival rate in the training cohort, (B) 1-, 3-, and 5-year survival rate in the validation cohort.

**Figure 6:**
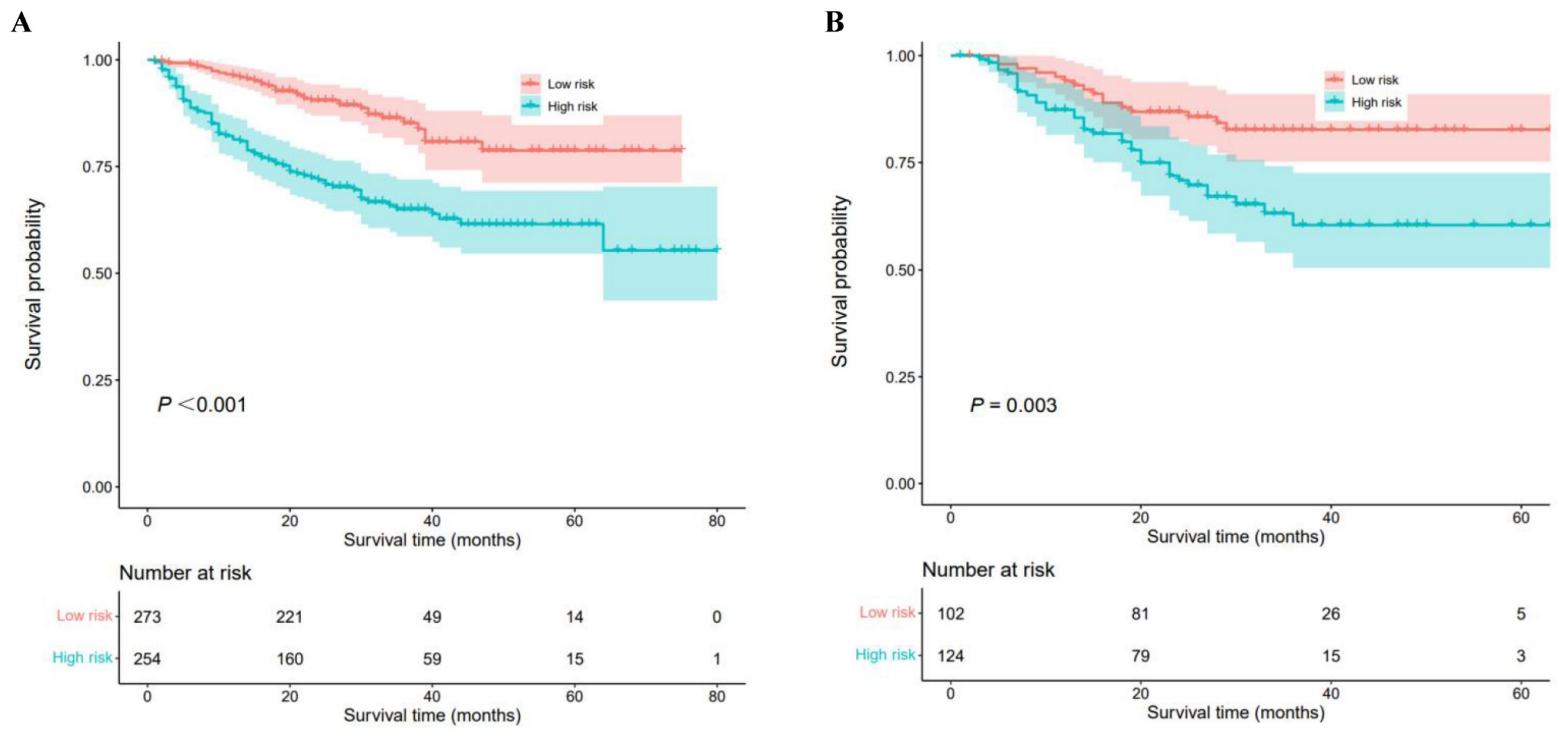
Kaplan-Meier analyses for overall survival of HCC patients with different risk groups. (A) Survival curves of HCC patients in the training cohort, (B) Survival curves of HCC patients in the validation cohort.

A nomogram was constructed to predict 1-, 3-, and 5-year OS, on the basis of the 6 variables described above (**Figure 3**). The Harrell's C-index for OS prediction was 0.723 (95% CI: 0.673-0.772) in the training cohort and 0.668 (95% CI: 0.588-0.748) in the validation cohort. The AUC value of the ROC analysis was 0.752 (95% CI: 0.683-0.821) in the training cohort and 0.747 (95% CI: 0.683-0.811) in the validation cohort (**Figure 4**). Overall, the calibration curves for 1-, 3-, and 5-year OS fitted well between the results from the prediction by the nomogram and the actual OS time in the training and the validation cohorts (**Figure 5**). Based on the median value of the total risk scores calculated by the nomogram, all patients could be stratified into the low-risk group (<2.80) and the high-risk group (≥2.80). In both the training and validation cohorts, the OS of HCC patients in the high-risk group was shorter than that in the low-risk group (**Figure 6**).

### The nomogram performed better in predicting OS in HCC patients than the AFP and CHI3L1

**Figure 7:**
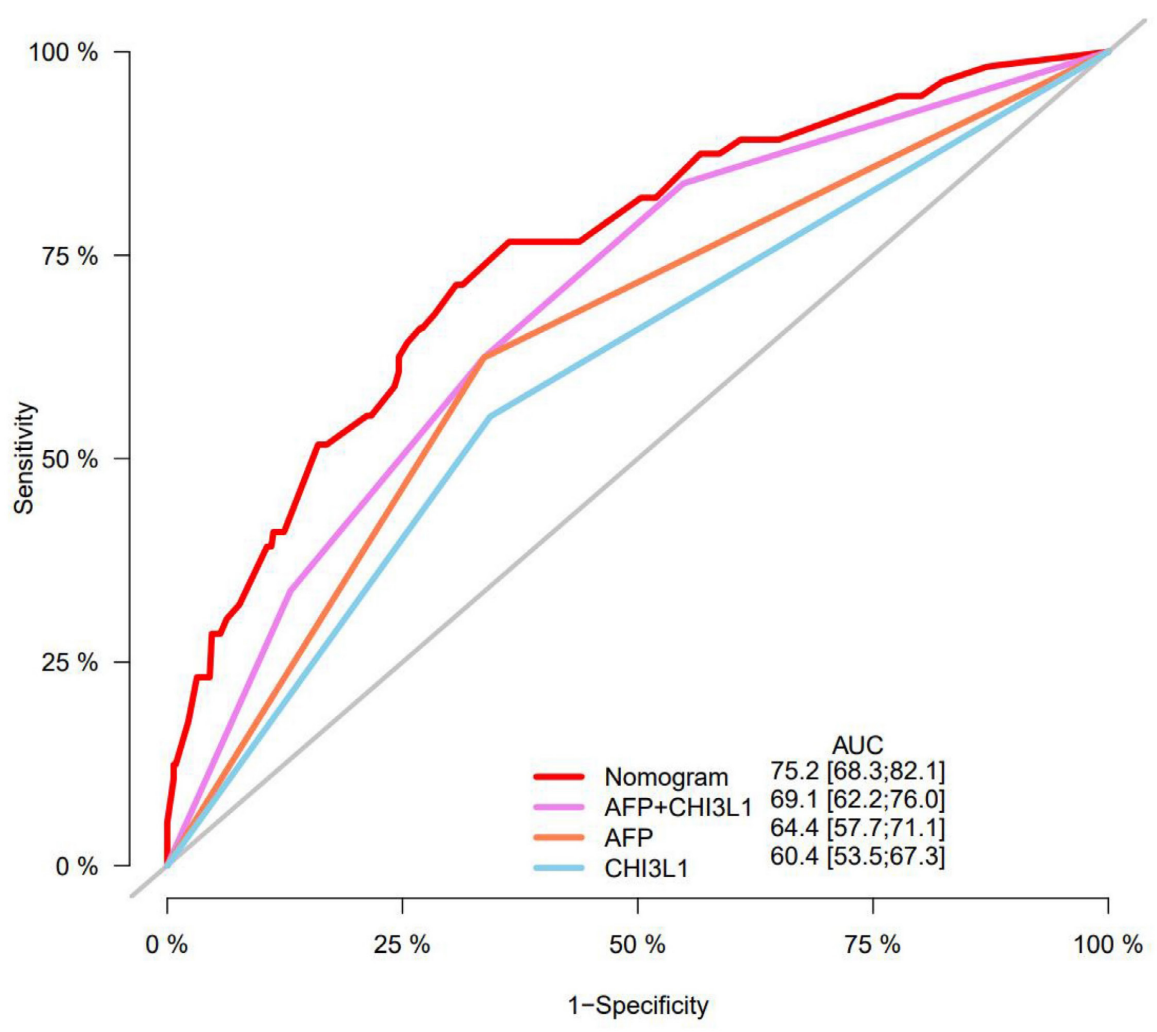
Predictive ability of the nomogram was compared with AFP and CHI3L1 by ROC curves.

Furthermore, predictive ability of the nomogram was compared with AFP and CHI3L1 by ROC curves (**Figure 7**). We found that the nomogram (AUC:0.752) performed better in predicting OS in patients with HCC than the AFP (AUC:0.644), and CHI3L1 (AUC:0.604).

## Discussion

Hepatocellular carcinoma is highly heterogeneous and has a high recurrence rate, resulting in poor survival rate after surgery. It is important to identify prognostic markers or simple prognostic models for better HCC patient management. Elevated serum CHI3L1 levels were found in patients with different pathological processes characterized by inflammation, fibrosis, and tissue remodeling 21. Studies have shown that deficiency in CHI3LI could improve liver fibrosis by promoting apoptosis and inhibiting the aggregation of liver macrophages 22. Qiu *et al.* found that the overexpression of CHI3L1 can intensify the proliferation, migration and invasion of HCC cells, and proposed a hypothesis that CHI3L1 may active TGF-β signaling pathway by binding to interleukin-13 receptor subunit α2 (IL-13Rα2) 23. In addition, it has been reported that elevated CHI3L1 accelerates HCC tumor progression by promoting ROS synthesis and inducing lipid peroxide (LPO) accumulation 24. In this study, we found that serum CHI3L1 level was associated with overall survival in HCC patients. The survival time of the HCC patients with low serum CHI3L1 levels was better than those with high serum CHI3L1 levels (*P* < 0.001). Univariate and multivariate Cox regression analyses indicated that CHI3LI was an independent predictor for OS in HCC. In addition, serum CHI3L1 expression was significantly associated with age, sex, tumor size, DCP, and NLR. These findings are consistent with those previously reported 17-18,25-27. Thus, we demonstrated that the serum CHI3L1 is a promising and valuable prognostic biomarker for patients with HCC after hepatic resection.

In the present study, we also confirmed previously reported risk factors for the prognosis in HCC patients: tumor size, AFP, DCP, INR, and NLR. Tumor size >5 cm was a significant prognostic factor in HCC, especially in HBV-associated patients 28. AFP is extensively studied as a biomarker for diagnosis and prognosis in patients with HCC. However, only about 10% of early HCC patients are known to have elevated AFP, suggesting that AFP alone is a limited prognostic factor 29. Even worse, there were about 38.1-39.4 % of HCC are AFP-negative, rendering AFP useless for these patients 30,31. The combination of serum CHI3L1 and AFP was able to predict outcomes of HCC patients undergoing TACE than either alone 26. DCP, also known as PIVKA-II, is an immature form of prothrombin that does not have any clotting function and is caused by an acquired defect in post-translational carboxylation of prothrombin precursors 32. Studies demonstrated that elevated DCP levels were associated with large tumor sizes and recurrences, poor differentiation, and intrahepatic metastasis in HCC patients 33,34. In addition, BALAD and BALAD2 scores derived from DCP, AFP, AFP-L3, albumin and bilirubin have been used as a prognostic system for HCC and have good application value 35,36. Previous studies have demonstrated a sex difference in the subgroup with lower INR and showed that the lower level of INR for male patients with HCC showed a favorable overall survival 37. Moreover, preoperative INR could predict the recurrence of early HCC after liver resection 38. However, the optimal cutoff value for INR in predicting the prognosis of HCC remains unclear. Our results suggest that INR≥1.05 may serve as the cutoff value for possible adverse postoperative outcomes in HCC patients. Consistent with our findings, a multicenter, multinational study found that high NLR values were associated with poor survival in HCC patients, and its combination with AFP is a useful prognostic marker for HCC 39.

Numerous nomograms have been developed and validated for predicting prognosis in various malignancies 40-42. Thus, we constructed a prognostic nomogram for further examine the clinical utility of CHI3L1 in HCC. The nomogram showed adequate discrimination and good consistency in the training cohort (C-index, 0.723; AUC: 0.752) and in the validation cohort (C-index, 0.668; AUC: 0.747). Based on the risk score, all patients could be divided into the low-risk group (<2.80) and the high-risk group (≥2.80). Kaplan-Meier curves revealed that the high-risk group of HCC patients had a poor OS (*P*<0.05). Interestingly, the prediction accuracy of the nomogram established in our study was superior to that of the single AFP and CHI3L1, which also proved that the combined model is more accurate and effective than single index model.

There are a few limitations to this study. First, the data was only collected from one institution, which could lead to selection bias; thus, the results need to be further verified by a prospective, multicenter study with a large sample size. Second, the follow-up samples after hepatectomy were not collected, which make it impossible to assess the effect of postoperative serum CHI3L1 on overall survival. Third, the findings may be subject to potential confounding due to lack of clinical characteristics, such as treatment methods, TNM stage, and vascular invasion.

## Conclusions

In summary, we identified that CHI3L1 may serve as a good predictor of a poor prognosis in HCC patients after hepatectomy. Moreover, the nomogram we constructed is a powerful tool for predicting HCC prognosis, superior to single AFP. The findings of our study should be further externally validation in other large, independent populations.

## Supplementary Material

Supplementary table.

## Figures and Tables

**Table 1:** Baseline characteristics of the study patients according to different CHI3L1 group

Characteristics	Overall	Low-CHI3L1 group	High-CHI3L1 group	*P* value
	(N=753)	(N =468)	(N =285)	
Age, n (%)				**<0.001**
<52	376 (49.9)	267 (57.1)	109 (38.2)	
≥52	377 (50.1)	201 (42.9)	176 (61.8)	
Sex, n (%)				**0.002**
female	93 (12.4)	44 (9.4)	49 (17.2)	
male	660 (87.6)	424 (90.6)	236 (82.8)	
Smoking, n (%)				0.296
No	423 (56.2)	256 (54.7)	167 (58.6)	
Yes	330 (43.8)	212 (45.3)	118 (41.4)	
Alcohol consumption, n (%)				0.142
No	461 (61.2)	277 (59.2)	184 (64.6)	
Yes	292 (38.8)	191 (40.8)	101 (35.4)	
BMI [kg/m^2^, n (%)]				0.812
18.5 ~ 25	247 (32.8)	155 (33.1)	92 (32.3)	
Others	506 (67.2)	313 (66.9)	193 (67.7)	
Hypertension, n (%)				0.485
No	653 (86.7)	409 (87.4)	244 (85.6)	
Yes	100 (13.3)	59 (12.6)	41 (14.4)	
Diabetes				0.814
No	698 (92.7)	433 (92.5)	265 (93.0)	
Yes	55 (7.3)	35 (7.5)	20 (7.0)	
Family history of liver cancer, n (%)				0.607
No	649 (86.2)	401 (85.7)	248 (87.0)	
Yes	104 (13.8)	67 (14.3)	37 (13.0)	
Liver Cirrhosis, n (%)				0.439
No	254 (33.7)	153 (32.7)	101 (35.4)	
Yes	499 (66.3)	315 (67.3)	184 (64.6)	
BCLC, n (%)				0.070
0-A	485 (64.4)	313 (66.9)	172 (60.4)	
B-C	268 (35.6)	155 (33.1)	113 (39.6)	
Child-Pugh grade, n (%)				0.370
A	726 (96.4)	449 (95.9)	277 (97.2)	
B	27 (3.6)	19 (4.1)	8 (2.8)	
Tumor number, n (%)				0.696
Solitary	600 (79.7)	375 (80.1)	225 (78.9)	
Multiple	153 (20.3)	93 (19.9)	60 (21.1)	
Tumor size [cm, n (%)]				**<0.001**
<5	278 (36.9)	199 (42.5)	79 (27.7)	
≥5	475 (63.1)	269 (57.5)	206 (72.3)	
HBeAg				0.963
Negative	40 (5.3)	25 (5.3)	15 (5.3)	
Positive	713 (94.7)	443 (94.7)	270 (94.7)	
HBsAg				0.968
Negative	130 (17.3)	81 (17.3)	49 (17.2)	
Positive	623 (82.7)	387 (82.7)	236 (82.8)	
AFP [ng/ml, n (%)]				0.239
<400	464 (61.6)	296 (63.2)	168 (58.9)	
≥400	289 (38.4)	172 (36.8)	117 (41.1)	
DCP [ng/ml, n (%)]				**<0.001**
<40	203 (27.0)	149 (31.8)	54 (18.9)	
≥40	550 (73.0)	319 (68.2)	231 (81.1)	
NLR				**0.003**
<3.34	633 (84.1)	408 (87.2)	225 (78.9)	
≥3.34	120 (15.9)	60 (12.8)	60 (21.1)	
INR				0.135
<1.05	503 (66.8)	322 (68.8)	181 (63.5)	
≥1.05	250 (33.2)	146 (31.2)	104 (36.5)	

BCLC, Barcelona Clinic Liver Cancer; AFP, alpha-fetoprotein; DCP, des-gamma-carboxy prothrombin; NLR, neutrophil-to-lymphocyte ratio; INR, international normalized ratio.

**Table 2:** Univariable and multivariable Cox regression analysis of variables associated with OS in study patients (N=753)

Variables	Univariate			Multivariable	
	HR (95%CI)	*P* value		HR (95%CI)	*P* value
Age (years)					
<52 vs. ≥52	0.84 (0.62-1.14)	0.264			
Sex					
Male vs. female	0.69 (0.46-1.04)	0.076			
Smoking					
No vs. Yes	1.18 (0.87-1.59)	0.291			
Alcohol consumption					
No vs. Yes	1.08 (0.791-1.46)	0.641			
BMI (kg/m^2^)					
Others vs. 18.5-25	1.23 (0.88-1.72)	0.220			
Hypertension					
No vs. Yes	0.69 (0.41-1.15)	0.154			
Diabetes					
No vs. Yes	0.96 (0.53-1.72)	0.886			
Family history of liver cancer					
No vs. Yes	1.07 (0.702-1.64)	0.745			
Liver cirrhosis					
No vs. Yes	1.14 (0.82-1.58)	0.429			
BCLC stage					
0-A vs. B-C	1.26 (0.93-1.72)	0.135			
Child-Pugh grade					
A vs. B	0.72 (0.30-1.76)	0.474			
Tumor number					
solitary vs. multiple	1.46 (1.03-2.05)	**0.032**		1.39 (0.98-1.95)	0.063
Tumor size (cm)					
<5 vs.≥5	2.22 (1.55-3.18)	**<0.001**		1.61 (1.10-2.35)	**0.013**
HBeAg					
Negative vs.Positive	0.78 (0.45-1.37)	0.391			
HBsAg					
Negative vs.Positive	1.17 (0.77-1.77)	0.466			
AFP (ng/ml)					
<400 vs. ≥400	2.08 (1.54-2.81)	**<0.001**		1.70 (1.25-2.31)	**<0.001**
DCP (ng/ml)					
<40 vs.≥40	2.39 (1.56-3.69)	**<0.001**		1.67 (1.06-2.62)	**0.026**
NLR					
<3.34 vs. ≥3.34	2.68 (1.93-3.74)	**<0.001**		2.07 (1.47-2.91)	**<0.001**
INR					
<1.05 vs. ≥1.05	1.72 (1.27-2.33)	**<0.001**		1.58 (1.16-2.14)	**0.003**
CHI3L1 (ng/ml)					
<198.94 vs. ≥198.94	1.77 (1.31-2.39)	**<0.001**		1.43 (1.05-1.94)	**0.024**

OS, overall survival; HR, hazard ratio; BCLC, Barcelona Clinic Liver Cancer; AFP, alpha-fetoprotein; DCP, des-gamma-carboxy prothrombin; NLR, neutrophil-to-lymphocyte ratio; INR, international normalized ratio.

**Table 3:** Univariable and multivariable Cox regression analysis of variables associated with OS in train cohort (N=527)

Variables	Univariate			Multivariable	
	HR (95%CI)	*P* value		HR (95%CI)	*P* value
Age (years)					
<52 vs. ≥52	0.98 (0.68-1.41)	0.926			
Sex					
Male vs. female	0.66 (0.41-1.06)	0.086			
Smoking					
No vs. Yes	1.02 (0.71-1.47)	0.912			
Alcohol consumption					
No vs. Yes	1.01 (0.69-1.46)	0.968			
BMI (kg/m^2^)					
Others vs. 18.5-25	1.26 (0.84-1.87)	0.264			
Hypertension					
No vs. Yes	0.77 (0.42-1.4)	0.394			
Diabetes					
No vs. Yes	0.94 (0.46-1.92)	0.861			
Family history of liver cancer					
No vs. Yes	1.01 (0.61-1.70)	0.957			
Liver cirrhosis					
No vs. Yes	1.11 (0.75-1.64)	0.594			
BCLC stage					
0-A vs. B-C	1.11 (0.76-1.62)	0.592			
Child-Pugh grade					
A vs. B	0.64 (0.21-2.03)	0.453			
Tumor number					
solitary vs. multiple	1.54 (1.00-2.37)	0.050		1.66 (1.08-2.57)	**0.022**
Tumor size (cm)					
<5 vs. ≥5	2.18 (1.43-3.34)	**<0.001**		1.61 (1.10-2.35)	**0.013**
HBeAg					
Negative vs. Positive	1.09 (0.52-2.29)	0.813			
HBsAg					
Negative vs. Positive	1.18 (0.71-1.98)	0.527			
AFP (ng/ml)					
<400 vs. ≥400	2.22 (1.54-3.20)	**<0.001**		1.97 (1.35-2.87)	**<0.001**
DCP (ng/ml)					
<40 vs. ≥40	2.36 (1.42-3.89)	**<0.001**		1.54 (0.91-2.62)	0.108
NLR					
<3.34 vs. ≥3.34	2.90 (1.95-4.31)	**<0.001**		2.34 (1.55-3.52)	**<0.001**
INR					
<1.05 vs. ≥1.05	1.56 (1.08-2.25)	**0.018**		1.45 (1.00-2.49)	**0.041**
CHI3L1 (ng/ml)					
<198.94 vs. ≥198.94	1.91 (1.32-2.75)	**<0.001**		1.46 (1.00-2.13)	**0.049**

OS, overall survival; HR, hazard ratio; BCLC, Barcelona Clinic Liver Cancer; AFP, alpha-fetoprotein; DCP, des-gamma-carboxy prothrombin; NLR, neutrophil-to-lymphocyte ratio; INR, international normalized ratio.
