# Supplementary material for: Serum CHI3L1 Levels Predict Overall Survival of Hepatocellular Carcinoma Patients after Hepatectomy
Source: J Cancer. 2024 Oct 14;15(19):6315–25. doi: 10.7150/jca.100791 (PMC11540517; doi:10.7150/jca.100791)
Supplement: Supplementary file 1 — Supplementary table. [file jcav15p6315s1.pdf]

Table S1. Baseline characteristics of patients with HCC in training cohort and validation cohort.

Characteristics	Overall (N=753)	Training cohort (N =527)	Validation cohort (N =226)	*P* value
Age, n (%)				0.855
＜52	376 (49.9)	262 (49.7)	114 (50.4)	
⩾52	377 (50.1)	265 (50.3)	112 (49.6)	
Sex, n(%)				0.983
female	93 (12.4)	65 (12.3)	28 (12.4)	
male	660 (87.6)	462 (87.7)	198 (87.6)	
Smoking, n (%)				0.626
No	423 (56.2)	293 (55.6)	130 (57.5）	
Yes	330 (43.8)	234 (44.4）	96 (42.5)	
Alcohol consumption, n (%)				0.116
No	461 (61.2)	313 (59.4)	148 (65.5)	
Yes	292 (38.8)	214 (40.6)	78 (34.5)	
BMI [kg/m^2, n (%)]				0.299
18.5 ~ 25	247 (32.8)	179 (34.0)	68 (30.1)	
Others	506 (67.2)	348 (66.0)	158 (69.9)	
Hypertension, n (%)				0.637
No	653 (86.7)	455 (86.3)	198 (87.6)	
Yes	100 (13.3)	72 (13.7)	28 (12.4)	
Diabetes				0.877
No	698 (92.7)	488 (92.6)	210 (92.9)	
Yes	55 (7.3)	39 (7.4)	16 (7.1)	
Family history of liver cancer, n (%)				0.61
No	649 (86.2)	452 (85.8)	197 (87.2)	
Yes	104 (13.8)	75 (14.2)	29 (12.8)	
Liver Cirrhosis, n (%)				0.969
No	254 (33.7)	178 (33.8)	76 (33.6)	
Yes	499 (66.3)	349 (66.2)	150 (66.4)	
BCLC, n (%)				0.209
0-A	485 (64.4)	347 (65.8)	138 (61.1)	
B-C	268 (35.6)	180 (34.2)	88 (38.9)	
Child-Pugh grade, n (%)				0.701
A	726 (96.4)	509 (96.6)	217 (96.0)	
B	27 (3.6)	18 (3.4)	9 (4.0)	
Tumor number, n (%)				0.017
Solitary	600 (79.7)	432 (82.0)	168 (74.3)	
Multiple	153 (20.3)	95 (18.0)	58 (25.7)	
Tumor size [cm, n (%)]				0.120
＜5	278 (36.9)	204 (38.7)	74 (32.7)	

≥5	475 (63.1)	323 (61.3)	152 (67.3)	
HBeAg				0.724
Negative	40 (5.3)	27 (5.1)	13 (5.8)	
Positive	713 (94.7)	500 (94.9)	213 (94.2)	
HBsAg				0.530
Negative	130 (17.3)	88 (16.7)	42 (18.6)	
Positive	623 (82.7)	439 (83.3)	184 (81.4)	
AFP [ng/ml, n (%)]				0.235
＜400	464 (61.6）	332 (63.0)	132 (58.4)	
≥400	289 (38.4）	195 (37.0)	94 (41.6)	
DCP [ng/ml, n (%)]				0.110
＜40	203 (27.0)	151 (28.7)	52 (23.0)	
≥40	550 (73.0)	376 (71.3)	174 (77.0)	
NLR				0.517
＜3.34	633 (84.1)	446 (84.6)	187 (82.7)	
≥3.34	120 (15.9）	81 (15.4)	39 (17.3)	
INR				0.862
＜1.05	503 (66.8)	351 (66.6)	152 (67.3)	
≥1.05	250 (33.2）	176 (33.4)	74 (32.7)	

BCLC, Barcelona Clinic Liver Cancer; AFP, alpha-fetoprotein; DCP, des-gamma-carboxy prothrombin; NLR, neutrophil-to-lymphocyte ratio; INR, international normalized ratio.
